# ACVIM consensus statement guidelines for the diagnosis, classification, treatment, and monitoring of pulmonary hypertension in dogs

**DOI:** 10.1111/jvim.15725

**Published:** 2020-02-17

**Authors:** Carol Reinero, Lance C. Visser, Heidi B. Kellihan, Isabelle Masseau, Elizabeth Rozanski, Cécile Clercx, Kurt Williams, Jonathan Abbott, Michele Borgarelli, Brian A. Scansen

**Affiliations:** ^1^ Department of Veterinary Medicine and Surgery, College of Veterinary Medicine University of Missouri Columbia Missouri; ^2^ Department of Medicine and Epidemiology, School of Veterinary Medicine University of California, Davis Davis California; ^3^ Department of Medical Sciences, School of Veterinary Medicine University of Wisconsin Madison Wisconsin; ^4^ Department of Sciences Cliniques, Faculté de Médecine Vétérinaire Université de Montréal Saint‐Hyacinthe Quebec Canada; ^5^ Department of Clinical Sciences, Cummings School of Veterinary Medicine Tufts University Medford Massachusetts; ^6^ Department of Clinical Sciences of Companion Animals and Equine University of Liège Liège Belgium; ^7^ Department of Pathobiology and Diagnostic Investigation, College of Veterinary Medicine Michigan State University East Lansing Michigan; ^8^ Department of Small Animal Clinical Sciences, College of Veterinary Medicine University of Tennessee Knoxville Tennessee; ^9^ Department of Small Animal Clinical Sciences Virginia Maryland College of Veterinary Medicine Blacksburg Virginia; ^10^ Department of Clinical Sciences Colorado State University Fort Collins Colorado

**Keywords:** echocardiography, pulmonary arterial hypertension, respiratory disease, tricuspid regurgitation velocity

## Abstract

Pulmonary hypertension (PH), defined by increased pressure within the pulmonary vasculature, is a hemodynamic and pathophysiologic state present in a wide variety of cardiovascular, respiratory, and systemic diseases. The purpose of this consensus statement is to provide a multidisciplinary approach to guidelines for the diagnosis, classification, treatment, and monitoring of PH in dogs. Comprehensive evaluation including consideration of signalment, clinical signs, echocardiographic parameters, and results of other diagnostic tests supports the diagnosis of PH and allows identification of associated underlying conditions. Dogs with PH can be classified into the following 6 groups: group 1, pulmonary arterial hypertension; group 2, left heart disease; group 3, respiratory disease/hypoxia; group 4, pulmonary emboli/pulmonary thrombi/pulmonary thromboemboli; group 5, parasitic disease (*Dirofilaria* and *Angiostrongylus*); and group 6, disorders that are multifactorial or with unclear mechanisms. The approach to treatment of PH focuses on strategies to decrease the risk of progression, complications, or both, recommendations to target underlying diseases or factors contributing to PH, and PH‐specific treatments. Dogs with PH should be monitored for improvement, static condition, or progression, and any identified underlying disorder should be addressed and monitored simultaneously.

**Abbreviations**
6MWT: 6‐minute walk test
BOAS: brachycephalic obstructive airway syndrome
C‐PH: combined postcapillary and precapillary pulmonary hypertension
CT: computed tomography
HFM: heart failure medications
Ipost‐PH: isolated postcapillary pulmonary hypertension
LA: left atrial
LHD: left‐sided heart disease
LHF: left‐sided heart failure
MMVD: myxomatous mitral valve disease
PAH: pulmonary arterial hypertension
PAP: pulmonary arterial pressure
PAWP: pulmonary arterial wedge pressure
PCH: pulmonary capillary hemangiomatosis
PDE5i: phosphodiesterase 5 inhibitor
PE/PT/PTE: pulmonary emboli/pulmonary thrombi/pulmonary thromboemboli
PG: pressure gradient
PH: pulmonary hypertension
POCUS: point‐of‐care ultrasound
PR: pulmonary regurgitation
Pre‐PH: precapillary pulmonary hypertension
PVD: pulmonary vascular disease
PVOD: pulmonary veno‐occlusive disease
PVR: pulmonary vascular resistance
RA: right atrial
RHC: right heart catheterization
RV: right ventricular
TRV: tricuspid regurgitation velocity

## 1 INTRODUCTION

This article is the report of the American College of Veterinary Internal Medicine consensus panel on pulmonary hypertension (PH) in dogs. The panel first established a working definition of PH and then proposed a clinically applicable classification scheme for PH in dogs; with this framework as the basis, practical guidelines for diagnostic, treatment, and monitoring recommendations were developed. Pulmonary hypertension is not a single disorder, and a multidisciplinary approach is optimal. Therefore, the consensus panel was comprised of board‐certified specialists in the areas of internal medicine, cardiology, emergency and critical care, diagnostic imaging, and anatomic pathology. The panel initially met in person to outline overall objectives and review the Delphi method^1^, ^2^ for consensus building. Review of the human and veterinary medical literature, development of electronic revisions, and conference calls were used to draft the document and, using a modification of the Delphi method, build consensus for recommendations. An advisory panel of 3 additional cardiologists helped develop echocardiographic definitions of PH and guidelines for echocardiographic assessment of PH. After development of a draft of the document, 2 additional advisory panel members were included to provide input and rate each of diagnostic, therapeutic, and monitoring recommendations. The final tally of numbers of panelists (out of 7) and outside experts (out of 5) agreeing with each recommendation was noted, with comments to clarify reasons for any dissent.

## 2 DEFINITION OF PH

### 2.1 Hemodynamic definitions and terminology

**Figure 1:**
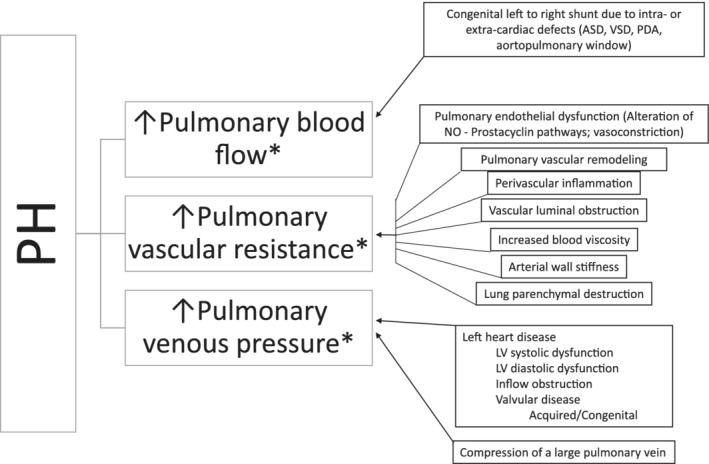
Development of pulmonary hypertension, defined as abnormally increased pressure within the pulmonary vasculature, results from increased pulmonary blood flow, increased pulmonary vascular resistance, increased pulmonary venous pressure, or some combination thereof. Normal pulmonary vasculature is comprised of thin‐walled arteries, veins and capillaries; a low pressure, low vascular resistance, and high capacitance system. In homeostasis, blood ejected from the right ventricle (RV) into the pulmonary trunk is directed to the right and left pulmonary arteries. The pulmonary arterial pressure remains low in homeostatic conditions by the dense network of pulmonary capillaries that can accommodate rapid transit of large volume of blood arriving from the pulmonary arteries. Following efficient gas exchange at the alveolar‐capillary interface, oxygenated blood is then collected by the pulmonary venules that unite to form the veins, which eventually open into the left atrium. Upon complex interactions of genetic and environmental factors, most of which are still poorly understood in dogs, homeostasis of pulmonary circulation can be disturbed. These disturbances (summarized in boxes) can lead to an excessive increase in pulmonary blood flow, increased pulmonary vascular resistance, or increased pulmonary venous pressure. Additionally, there is interplay between these factors*: increased pulmonary blood flow or increased pulmonary venous pressure can lead to increased pulmonary vascular resistance due to pulmonary arterial vasoconstriction, pulmonary vascular disease/remodeling, or both. When the average pulmonary arterial pressure increases above a certain threshold (25 mm Hg is commonly used in humans), PH results. With sustained PH, the RV has to work harder against increases in pulmonary pressures to move blood through the pulmonary vasculature. As a consequence, the RV undergoes structural alterations. Over time, the progressive increase in RV workload can ultimately lead to RV dysfunction and failure, resulting in heart failure (ascites), low output signs, and death. ASD, atrial septal defect; EDD, endothelial‐dependent dilatation; LV, left ventricle; NO, nitric oxide; PDA, patent ductus arteriosus; PH, pulmonary hypertension; VSD, ventricular septal defect

Pulmonary hypertension is defined by abnormally increased pressure within the pulmonary vasculature. The criterion standard method for diagnosis of PH is direct assessment of pulmonary arterial pressure (PAP) by right heart catheterization (RHC) and, in humans, PH has been defined as a mean PAP ≥25 mm Hg at rest.^3^ A schematic outlining the pathophysiology of PH is shown in Figure 1. Increased PAP is not a defining characteristic of a specific clinical condition but rather an abnormal hemodynamic state associated with numerous, diverse disorders.^4^ Increased PAP, in the absence of increased pulmonary vascular resistance (PVR), has several causes requiring different management strategies and having different outcomes, including increased cardiac output, left‐to‐right shunts, and increased pulmonary arterial wedge pressure (PAWP) secondary to the left‐sided heart disease (LHD). Increased PAP associated with increased PAWP (>15 mm Hg in humans^5^; a surrogate for left atrial [LA] or left ventricular filling pressure), is referred to as postcapillary PH. Postcapillary PH occurs most commonly in dogs with LHD that have increased LA pressure. It develops because LA hypertension increases the load on the right ventricle and indirectly necessitates the development of higher systolic right ventricular (RV) pressures. Chronic postcapillary PH can lead to pulmonary arterial vasoconstriction and pulmonary vascular disease (PVD), which increase PVR.^6^, ^7^ Thus, postcapillary PH can occur in isolation (isolated postcapillary PH [Ipost‐PH]) or can occur together with increased PVR (combined postcapillary and precapillary PH; C‐PH) as a result of chronic, progressive LHD. In humans, C‐PH currently is defined by a pressure difference of ≥7 mm Hg between diastolic PAP and PAWP.^5^ An equivalent pressure difference has not been established in dogs. Increased PAP also can be caused by increased PVR and structural pulmonary arterial changes associated with PVD due to a variety of other causes. Increased PAP associated with increased PVR in the absence of increased LA pressure defines precapillary PH (pre‐PH).

## 3 ECHOCARDIOGRAPHIC ASSESSMENT OF PH

Echocardiography should be viewed as a clinical tool to help assess the probability that a dog has PH, rather than a definitive diagnostic test for the presence of PH. Definitive diagnosis of PH requires RHC. Echocardiographic assessment of PH is based largely on characteristic cardiac changes that occur secondary to PH (ie, echocardiographic signs of PH) and by estimating PAP from spectral Doppler tracings. Because RHC rarely is utilized for definitive diagnosis of PH in dogs, veterinarians rely on echocardiography for diagnosis, classification, and management of dogs with PH.

However, clinicians should be mindful of limitations of the echocardiographic examination (particularly Doppler echocardiography) and of inaccuracy,^8^ variability, and imprecision^9^, ^10^ potentially encountered when using echocardiography to estimate PAP in individual dogs.

The panel has kept these echocardiographic shortcomings in mind when proposing guidelines and recommendations. Because many echocardiographic signs of PH have not been compared to RHC findings in dogs, we have consulted current echocardiographic guidelines for humans^11^, ^12^ and the veterinary medical literature when relevant citations were available to establish these guidelines. The proposed criteria are intended to avoid misdiagnosis and inappropriate treatment of PH that might have lasting impact on the dog and its owner. Lastly, echocardiographic assessment of PH is just 1, albeit important, aspect in the overall clinical assessment of a dog with suspected PH. Echocardiographic findings always should be interpreted within the context of other clinical findings, especially the presence or absence of clinical signs suggestive of PH (Table 1) and right‐sided heart failure status, as well as results of concurrent diagnostic testing.

**Table 1:** Clinical findings suggestive of pulmonary hypertension (PH) in dogs^a^

Findings strongly suggestive of PH	Findings possibly suggestive of PH
Syncope (especially with exertion or activity) without another identifiable cause	Tachypnea at rest
Respiratory distress at rest	Increased respiratory effort at rest
Activity or exercise terminating in respiratory distress	Prolonged postexercise or post‐activity tachypnea
Right‐sided heart failure (cardiogenic ascites)	Cyanotic or pale mucous membranes

a It should be noted that none of these clinical signs are specific solely for PH and therefore other causes of clinical signs are not excluded. Although these clinical signs may be due to underlying respiratory disease, more pronounced clinical signs reflect more severe disease, with more severe disease likely to result in PH.

### 3.1 Echocardiographic signs of PH and estimating PAP

The use of Doppler echocardiography to estimate PAP is crucial to the echocardiographic assessment of dogs with suspected PH. Assuming the absence of RV outflow tract obstruction (eg, pulmonary valve stenosis), estimating systolic PAP involves quantifying peak tricuspid regurgitation velocity (TRV), and then derivation of the pressure gradient (PG) between the RV and right atrium using the simplified Bernoulli Equation (PG = 4 × velocity [m/s]^2^). An estimate of right atrial (RA) pressure is added to the calculated PG to yield estimated systolic PAP. Validated methods to estimate RA pressure are unavailable in dogs, and therefore estimates of RA pressure are arbitrary and potentially flawed.^8^, ^10^, ^13^ Consequently, we recommend using only continuous wave Doppler measurement of TRV (versus estimated systolic PAP) as a key metric in determining PH probability as long as clinicians are aware that systolic PAP might be underestimated when severe RA hypertension is present. Measured TRV depends upon many factors including RV function and pericardial restraint^14^ in addition to PAP and PVR. Additionally, poor patient cooperation and labored respiration affect measurements. Accurate interpretation of a TRV signal includes consideration of all factors impacting RV systolic pressure. A pulmonary regurgitation (PR) jet also can be used to estimate mean or diastolic PAP.^11^, ^15^, ^16^ Applying the simplified Bernoulli equation to spectral Doppler measurements of peak diastolic PR velocity provides an estimate of mean PAP.^16^ Similarly, peak diastolic PR jet velocity offers an estimate of diastolic PAP after adding an estimate of RA pressure.^15^ Because tricuspid regurgitation has been utilized more commonly, and clinicians conventionally have viewed PH in relation to estimates of systolic PAP, TRV can be considered the primary metric to estimate PAP and the key component to determine the probability of PH in at‐risk dogs. Recommended echocardiographic criteria used to help determine the probability of PH are presented in Table 2.

**Table 2:** Echocardiographic probability of PH in dogs

Peak tricuspid regurgitation velocity (m/s)	Number of different anatomic sites of echo signs of PH^a^	Probability of PH
≤3.0 or not measurable	0 or 1	Low
≤3.0 or not measurable	2	Intermediate
3.0 to 3.4	0 or 1	Intermediate
>3.4	0	Intermediate
≤3.0 or not measurable	3	High
3.0 to 3.4	≥2	High
>3.4	≥1	High

a See Table 3.

**Figure 2:**
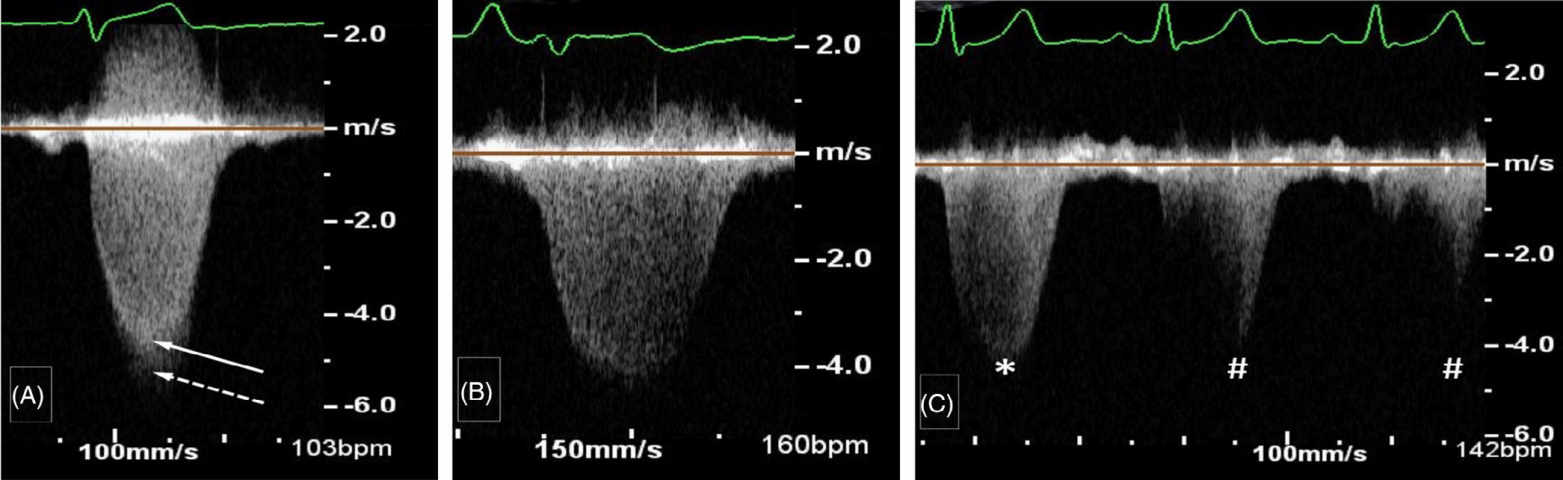
Example measurements of tricuspid regurgitation velocity (TRV) spectra acquired using continuous wave Doppler. A, The solid arrow shows the recommended measurement of TRV. The dense outer edge of the velocity profile (brighter signal) is measured and measurement sof the fine linear signals has been avoided. The dotted arrow represents a measurement that includes the fine linear signals or “feathered edge,” which likely overestimates TRV. B, A TRV signal of good quality is shown. Notice the envelope is fully visible, the signal is not overgained (helps to avoid fine linear signals), the sweep speed is increased, and the scale along the vertical axis is nearly filled by the TRV signal. C, Shows 3 TRV signals, 1 of moderate quality (*) that is not completely filled in but can be measured by extrapolation and 2 others of poor quality (#) that are unreliable and should not be measured

Although specific techniques of echocardiographic image acquisition and measurement are beyond the scope of this consensus statement, Doppler spectra should be well visualized and their shape and contour should comply with known hemodynamic principles. Care should be taken to align the cursor parallel to the direction of flow, optimize gain settings, and to measure regurgitant jets at the dense outer edge of the velocity profile while avoiding measurement of fine linear signals, also called the “feathered edge” (Figure 2).^29^, ^30^, ^31^ Because tricuspid regurgitation jets can be eccentric, nonstandard imaging planes might be necessary for visualization of tricuspid regurgitation and acquisition of TRV.

In addition to TRV, several echocardiographic signs of PH aid probability assessment for PH. These signs involve 3 anatomic sites: (1) ventricles, (2) pulmonary artery (although pulmonary artery is the commonly used term in clinical practice, the preferred anatomic term for the main pulmonary artery is pulmonary trunk^32^ and herein we consider them synonymously), and (3) RA and caudal vena cava (Table 3). These echocardiographic signs of PH largely involve assessment of the ventricles (left ventricular and RV size and remodeling,^19^, ^21^ systolic flattening of the interventricular septum,^17^ and RV systolic function^19^, ^23^, ^24^, ^25^, ^26^, ^27^, ^33^), the pulmonary artery (pulmonary artery size^17^, ^18^, ^19^ and flow profile,^13^, ^17^, ^28^, ^34^), and the RA and caudal vena cava (RA^19^, ^21^ and caudal vena cava size^19^). Unfortunately, there is a lack of agreement on how best to assess (ie, measure and quantify) these echocardiographic variables in dogs with PH, and specific echocardiographic guidelines are beyond the scope of this consensus statement.

**Table 3:** Anatomic sites of echocardiographic signs of PH used to help assess the probability of PH in dogs

Anatomic site 1: Ventricles	Anatomic site 2: Pulmonary artery	Anatomic site 3: Right atrium and caudal vena cava
Flatting of the interventricular septum (especially systolic flattening)	Pulmonary artery enlargement (PA/Ao >1.0^17^, ^18^)	Right atrial enlargement^19^, ^20^
Underfilling or decreased size of the left ventricle^a^	Peak early diastolic PR velocity >2.5 m/s	Enlargement of the caudal vena cava^19^
Right ventricular hypertrophy (wall thickening, chamber dilation, or both)^19^, ^21^	RPAD index <30%^17^, ^22^	
Right ventricular systolic dysfunction^19^, ^23^, ^24^, ^25^, ^26^, ^27^	RV outflow Doppler acceleration time (<52‐58 ms) or acceleration time to ejection time ratio (<0.30)^17^, ^18^, ^28^	
	Systolic notching of the Doppler RV outflow profile (caution: false positives are possible)	

Abbreviations: PR, pulmonary regurgitation; RPAD, right pulmonary artery distensibility; RV, right ventricular.

a Not applicable for dogs with group 2 PH due to the confounding effects of LV remodeling secondary to LHD.

### 3.2 Echocardiographic probability of PH with LHD

Recommendations in Tables 2 and 3 for echocardiographic assessment of PH pertain to the use of a probability‐based approach for the assessment PH for all causes of PH (groups 1‐6, Table 4). In addition to criteria in Tables 2 and 3, identifying dogs with PH secondary to LHD requires fulfilling 2 additional echocardiographic criteria: (1) documentation of LHD (eg, mitral or aortic valvular disease or left ventricular dysfunction) and (2) unequivocal LA enlargement. Because RHC and PAWP determination are performed rarely, LA enlargement is utilized as a crude, but robust, surrogate for chronically increased PAWP and postcapillary PH. By definition, patients with PH secondary to LHD must have increased PAWP.^5^ The panel recognizes it is possible to encounter acutely increased PAWP, and therefore iPost‐PH secondary to LHD without LA enlargement caused by, for example, ruptured chordae tendineae in a dog with myxomatous mitral valve disease (MMVD). Sonographers should be cognizant of this uncommon exception. Chronically increased PAWP is a necessary component of clinically relevant C‐PH secondary to LHD.^5^, ^6^ In other words, it is less likely for PH, and particularly C‐PH, to be secondary to LHD unless unequivocal LA enlargement also is identified. Echocardiographic signs of PH such as decreased LV size or LV underfilling are not applicable to dogs with PH secondary to LHD because of the confounding effects of LV remodeling secondary to LHD. In dogs with PH secondary to LHD, criteria proposed in Tables 2 and 3 for high probability of PH are more likely to identify patients with C‐PH. This approach is supported by clinical studies of dogs with MMVD demonstrating the clinical and prognostic relevance of TRV >3.5‐3.7 m/s.^82^, ^152^

**Table 4:** Proposed classification of pulmonary hypertension in the dog^a^

**Figure 3:**
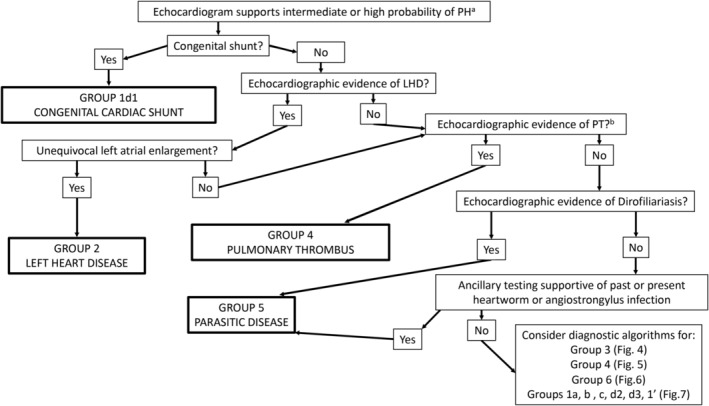
Algorithm demonstrating the overall diagnostic approach to the 6 groups of pulmonary hypertension in which echocardiography performed early in the clinical evaluation identifies intermediate or high probability PH. In addition to determining an intermediate or high probability of PH, echocardiography can also be used to support, confirm, or refute pathology in group 1d1, group 2, group 4, and group 5. The order in which diagnostic algorithms should be consulted are group 3 (Figure 4) and group 4 (Figure 5) with group 5 potentially being identified on this initial algorithm or within the group 3 algorithm. The group 1 algorithm (Figure 7) generally used after ruling out disorders in groups 2‐6. Critical to appropriate use of the diagnostic algorithms is the understanding that dogs frequently have greater than 1 type of pathology contributing to PH either across groups (eg, a dog with MMVD with interstitial lung disease is encompassed in groups 2 and 3, respectively) or within a group (eg, a dog with tracheal collapse and fibrotic lung disease both fall within group 3). Clinical evaluation must drive the diagnostic approach and make sense in context of localizing disease and pursuit of comorbid conditions. For example, a small breed dog with left‐sided heart failure that has inspiratory stridor in addition to rapid, shallow breathing should not have the diagnostic algorithm terminated after diagnosis of group 2c1a disease; instead, further evaluation for an upper airway defect such as extrathoracic tracheal collapse should be pursued. ^a^Thoracic radiographs are frequently obtained before echocardiography and may provide additional findings supportive of underlying PH etiology. ^b^Evidence of an in situ PT in the main pulmonary artery may be noted on echocardiographic examination. LHD, left‐sided heart disease; PH, pulmonary hypertension, PT, pulmonary thrombus

**Figure 4:**
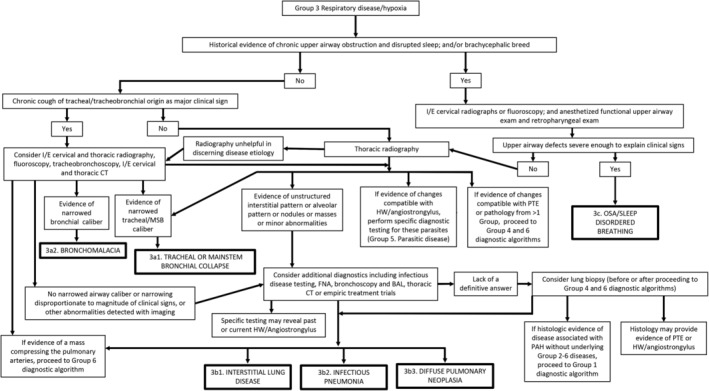
Diagnostic algorithm for discrimination of group 3 respiratory disease/hypoxia in dogs. Proper interpretation relies on confirmation that the comprehensive clinical picture can be explained solely by the “final diagnosis” (bold boxes); otherwise, continue to evaluate for PH in other subcategories of group 3 and in groups 1, 4, 5, and 6. BAL, bronchoalveolar lavage; CT, computed tomography; FNA, fine‐needle aspiration; HW, heartworm; I/E, paired inspiratory/expiratory series; MSB, mainstem bronchial; OSA, obstructive sleep apnea; PAH, pulmonary arterial hypertension; PH, pulmonary hypertension; PTE, pulmonary thromboembolism

**Figure 5:**
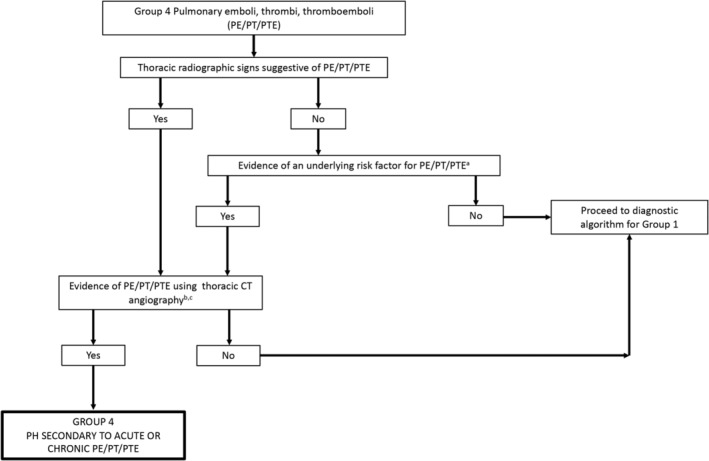
Diagnostic algorithm for discrimination of group 4 pulmonary emboli/thrombi/thromboemboli in dogs. Proper interpretation relies on confirmation that the comprehensive clinical picture can be explained solely by the “final diagnosis” of PE/PT/PTE; otherwise, continue to evaluate for PH in groups 1 and 6. ^a^Risk factors include but are not limited to hypercoagulability (eg, CBC, serum biochemical profile, UA, UP:C, TEG, D‐dimers), pulmonary arterial mass, endothelial injury (eg, IV catheter, polytrauma with immobility), and evidence of air or fat emboli. ^b^Ideally triphasic angiography is recommended. ^c^Ventilation‐perfusion scans using nuclear scintigraphy can also be used to document PE/PT/PTE but are not commonly performed. CT, computed tomography; PH, pulmonary hypertension, UA, urinalysis; UP:C, urine protein:creatitine ratio; TEG, thromboelastography

**Figure 6:**
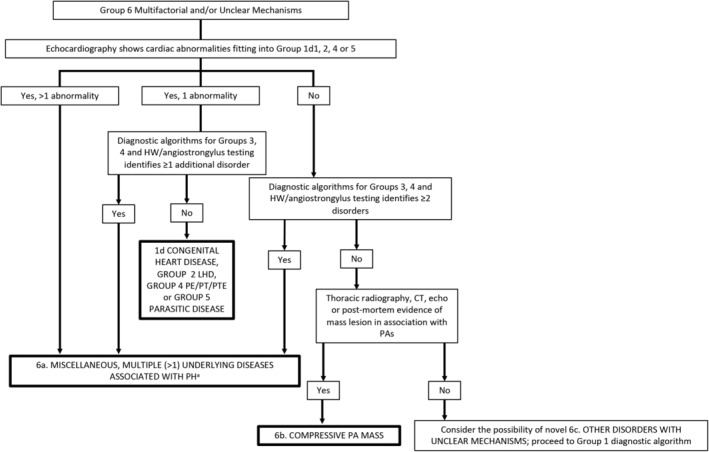
Diagnostic algorithm for determination of group 6 (multifactorial and/or unclear mechanisms) in dogs. Confirm that the comprehensive clinical picture can be explained solely by the “final diagnosis” (bold boxes). Otherwise, consider hematologic, systemic, and metabolic disorders of unclear mechanism that have been identified in humans with PH^148^ and if not present or likely to be causative of PH, continue to evaluate for PH in group 1. Each disease identified must be addressed in the overall treatment plan. ^a^Each will need to be addressed independently when considering optimal treatment. CT, computed tomography; HW, heartworm; LHD, left heart disease; PA, pulmonary artery; PH, pulmonary hypertension; PE/PT/PTE, pulmonary emboli/thrombi/thromboemboli

**Figure 7:**
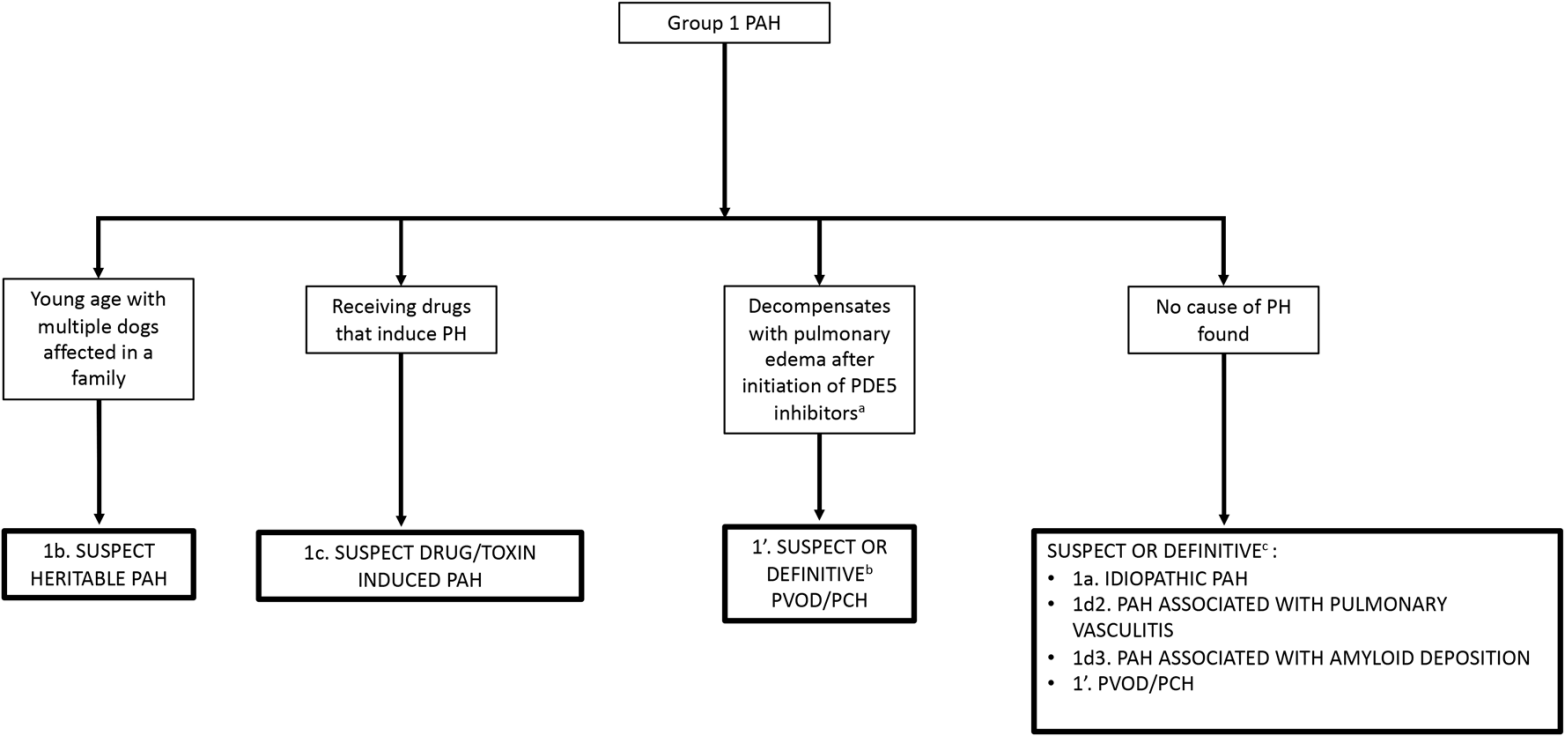
Diagnostic algorithm for discrimination of group 1 pulmonary arterial hypertension in dogs. The approach to group 1 disorders generally requires ruling out groups 2‐6 disorders first. Importantly, histologic changes associated with the pulmonary vasculature in group 1a‐c are not pathognomonic and can occur secondary to primary cardiac and respiratory disease. Histopathology can provide definitive diagnosis for group 1d2, 1d3, and 1′ disorders. ^a^Extrapolated from humans; not definitively proven for canine PVOD/PCH. ^b^Confirmed on histopathology. PAH, pulmonary arterial hypertension; PH, pulmonary hypertension, PDE5, phosphodiesterase 5; PVOD/PCH, pulmonary veno‐occlusive disease/pulmonary capillary hemangiomatosis

**1. Pulmonary arterial hypertension**
1a. Idiopathic (IPAH)^35^, ^36^, ^37^, ^38^, ^39^^,^ b ^;^ 40, 41, 42, 43, 44, 45 ^,^ c
1b. Heritable^46^, ^47^
1c. Drugs and toxins induced^48^, ^49^^,^ d
1d. Associated with (APAH):
1d1. Congenital cardiac shunts^17^, ^18^, ^35^, ^41^, ^42^, ^43^, ^46^, ^47^, ^50^, ^51^, ^52^, ^53^, ^54^, ^55^, ^56^, ^57^, ^58^, ^59^, ^60^, ^61^, ^62^, ^63^, ^64^, ^65^, ^66^, ^67^, ^68^, ^69^, ^70^, ^71^, ^72^, ^73^, ^74^, ^75^
1d2. Pulmonary vasculitis^63^, ^71^, ^76^, ^77^, ^78^
1d3. Pulmonary vascular amyloid deposition^79^
**1′. Pulmonary veno‐occlusive disease (PVOD) or pulmonary capillary hemangiomatosis (PCH)** 80, 81 ^,^ e
**2. Pulmonary hypertension due to left heart disease**
2a. Left ventricular dysfunction
2a1. Canine dilated cardiomyopathy^18^, ^25^, ^35^, ^43^, ^50^, ^51^
2a2. Myocarditis^35^
2b. Valvular disease
2b1. Acquired
2b1a. Myxomatous mitral valve disease^10^, ^13^, ^17^, ^18^, ^20^, ^24^, ^25^, ^34^, ^35^, ^41^, ^42^, ^43^, ^50^, ^51^, ^52^, ^82^, ^83^, ^84^, ^85^, ^86^, ^87^, ^88^, ^89^
2b1b. Valvular endocarditis
2c1. Congenital/acquired left heart inflow/outflow tract obstruction and congenital cardiomyopathies
2c1a. Mitral valve dysplasia^18^, ^52^
2c2a. Mitral valve stenosis^90^
2c3a. Aortic stenosis^43^
**3. Pulmonary hypertension secondary to respiratory disease, hypoxia or both** f
3a. Chronic obstructive airway disorders^g^
3a1. Tracheal or mainstem bronchial collapse^18^, ^85^, ^91^
3a2. Bronchomalacia^91^, ^92^, ^93^
3b. Primary pulmonary parenchymal disease
3b1. Interstitial lung disease (reviewed in^94^, ^95^)
3b1a. Fibrotic lung disease^18^, ^28^, ^40^, ^91^, ^92^, ^96^
3b1b. Cryptogenic organizing pneumonia/secondary organizing pneumonia^97^
3b1c. Pulmonary alveolar proteinosis^98^
3b1d. Unclassified interstitial lung disease (ILD)^41^, ^66^, ^85^, ^91^, ^99^, ^100^
3b1e. Eosinophilic pneumonia/eosinophilic bronchopneumopathy^66^, ^92^, ^101^
3b2. Infectious pneumonia^h^: Pneumocystis^91^, ^102^, ^103^; Ehrlichia?^104^, ^105^
3b3. Diffuse pulmonary neoplasia^35^, ^66^, ^85^, ^91^, ^92^, ^106^
3c. Obstructive sleep apnea/sleep disordered breathing^107^, ^108^^,^ d ^;^ 91, 92, 109 ^,^ i
3d. Chronic exposure to high altitudes^110^, ^111^, ^112^
3e. Developmental lung disease^91^
3f. Miscellaneous: bronchiolar disorders^91^; bronchiectasis^91^; emphysema^91^, ^113^; pneumonectomy^114^
**4. Pulmonary emboli/thrombi/thromboemboli (PE/PT/PTE)** 115, 116, 117, 118, 119, 120, 121
4a. Acute PE/PT/PTE^8^, ^122^, ^123^, ^124^, ^125^,^d^
(Massive PE/PT/PTE with RV dysfunction or submassive PE/PT/PTE without RV dysfunction)
4b. Chronic PE/PT/PTE^126^,^d^^,^ 40
**5. Parasitic disease (*Dirofilaria* or *Angiostrongylus* infection** 17, 25, 35, 41, 44, 50, 51, 66, 127, 128, 129, 130, 131, 132, 133, 134, 135, 136, 137, 138, 139, 140, 141, 142, 143, 144, 145, 146, 147 **)** j
**6. PH with multifactorial or unclear mechanisms** k
6a. Disorders having clear evidence of 2 or more underlying groups 1‐5 pathologies contributing to PH^l^
6b. Masses compressing the pulmonary arteries (eg, neoplasia, fungal granuloma, etc.)
6c. Other disorders with unclear mechanisms

a Given the limitations of the veterinary literature (eg, single case reports or small case series, retrospective study design, frequent presence of confounding comorbid conditions contributing to PH, lack of uniform and rigorous diagnostic testing to definitively rule out comorbid conditions, among others), not all panelists agree with provided references to support the disease as the cause of PH. Larger, prospective carefully designed studies will be required to provide the necessary evidence to further refine this classification scheme.

b In the veterinary literature, when no underlying cause of PH has been found, PH is often assumed to be “idiopathic.” However, it is important to recognize the difference between not finding a cause after an exhaustive diagnostic evaluation and calling a disease idiopathic after a cursory evaluation (see Figures 3, 4, 5, 6, 7). The first 5 references are considered definitive studies as histopathology documents a pulmonary arteriopathy in the absence of a known cause.

c The next 6 references are considered questionable support for IPAH; although no identified cause was found, the diagnostic evaluation may not have been reported or have been incomplete and histologic evaluation was not performed.

d Experimental canine studies.

e PVOD and PCH can occur in tandem.

f In the peer‐reviewed veterinary literature, many studies refer to “chronic respiratory/pulmonary disease” or “idiopathic” respiratory disease, or “chronic tracheobronchial disease” without definitive documentation of the specific underlying disorder.^35^, ^40^, ^41^, ^42^, ^66^, ^85^, ^149^ Other listed “definitive” diagnoses may be published without ruling out disease mimics in an exhaustive fashion (eg, thoracic radiography alone can be definitive for collapsing trachea but nondefinitive for bronchomalacia or fibrotic lung disease). Without a criterion standard definitive confirmation (eg, bronchoscopy for bronchomalacia or lung biopsy for pulmonary fibrosis), many of these respiratory diseases are likely inadequately characterized. Additionally, many dogs with disorders associated with PH in humans do not get a specific evaluation for PH; thus the group 3 disorders are likely grossly underestimated. Additionally, disorders which are not clearly documented or are undocumented to cause PH in the dog include pharyngeal collapse,^150^ laryngeal collapse, laryngeal paralysis, and epiglottic retroversion.

g Although “chronic bronchitis” has been listed as a diagnosis in some canine reports,^18^, ^85^ this syndrome alone in the dog is unlikely to cause PH. The term chronic obstructive pulmonary disease (COPD) used in humans encompasses underlying and overlapping conditions such as chronic bronchitis and emphysema. Both cause airflow limitation and dyspnea in people. Canine chronic bronchitis by itself (ie, without concurrent bronchomalacia) does not cause airflow limitation leading to increased expiratory respiratory effort and emphysema is very rare in dogs, thus the term COPD is inappropriate to use in this species. Tracheal and mainstem bronchial collapse and bronchomalacia are common causes of obstructive airway disorders; however, referenced studies proving they cause PH are somewhat limited by many reported dogs having comorbid conditions also known to cause PH.

h *Angiostrongylus* and *Dirofilaria* are excluded from infectious causes of pneumonia as the pathophysiology of PH is usually multifactorial with these parasitic infections. The term “pneumonia” by itself does not necessarily imply an infectious etiology and care must be taken when interpreting results of studies that do not specifically identify an organism but find compatible radiographic changes or inflammatory cells on airway lavage or histopathology.^35^, ^51^, ^66^ These cases may represent ILDs.

i Brachycephalic obstructive airway syndrome is listed under obstructive sleep apnea/sleep disordered breathing as the dog is a model for human disease.^151^ However, as this is a heterogeneous syndrome with multiple defects, clinical manifestations could also be classified under chronic obstructive airway disorders.

j *Dirofilaria* and *Angiostrongylus* have been associated with endarteritis,^17^, ^25^, ^35^, ^41^, ^44^, ^50^, ^51^, ^66^, ^127^, ^128^, ^129^, ^130^, ^131^, ^132^, ^133^, ^134^, ^135^, ^136^, ^137^, ^138^, ^139^, ^140^, ^141^, ^142^ PE/PT/PTE,^147^ inflammatory pulmonary parenchymal disease,^143^, ^144^, ^145^, ^146^ or all, as their mechanisms of PH.

k In humans, hematologic disorders (eg, certain types of anemia, myeloproliferative disorders, and splenectomy), systemic disorders with lung involvement (eg, sarcoidosis, Langerhans cell histiocytosis, vasculitis, etc), metabolic disorders (disorders of impaired cell metabolism, thyroid disease), and other diseases not well classified in another group (eg, compressive lesions such as lymphadenopathy, tumor or fibrosing mediastinitis obstructing the pulmonary arteries, etc) comprise the multifactorial, unclear mechanism group, or both.^148^ As these analogous disorders with rare exception either do not occur in dogs or if they occur, may not be documented to cause PH, additional research and modification of these group 6 disorders in dogs will likely be needed. This is a particularly poorly understood category and it is likely that other diseases will be added in the future with additional investigation.

l To be classified as 6a, there must be identified diseases in more than 1 of the group 1‐5 disorders (eg, group 2 MMVD and group 3b1 ILD) and not just 2 or more types of disease within a single disorder (group 3a1 tracheal collapse and group 3b1a fibrotic lung disease).

### 3.3 Proposed clinical definition of PH

In the veterinary medical literature, the degree or severity of PH has been classified as mild, moderate, or severe. These categories are based on the PG derived from TRV (also called the tricuspid regurgitation PG) or estimated systolic PAP. Cutoffs used for these categories (mild, moderate, severe) are arbitrary, and the categories are potentially misleading or flawed. For example, based on the conventional definition of PH (based solely on estimated systolic PAP), a dog with severe PH can be free of clinical signs, whereas a dog with moderate PH could be in right‐sided heart failure. Therefore, the panel does not advocate their use. Instead, severity of PH should be based on clinical findings (Table 1) and outcome data from large (prospective) longitudinal studies, which unfortunately are unavailable. The panel recognizes the probabilistic approach to diagnosis of PH presents challenges, particularly regarding standardized enrollment in future clinical studies of dogs with suspected PH. Therefore, we propose that the clinical definition of PH should include dogs with intermediate or high probability of PH, and specifically, a tricuspid regurgitation PG cutoff of >46 mm Hg (TRV >3.4 m/s). This roughly equates to what has been conventionally referred to as at least moderate PH. Until there are echocardiographic variables that are repeatable and have acceptable diagnostic accuracy relative to RHC, this cutoff value could be used with an understanding of its limitations.

## 4 COMPREHENSIVE CLINICAL ASSESSMENT FOR PH

Diagnosis of PH requires comprehensive evaluation including consideration of signalment, clinical signs, echocardiographic parameters supporting PH, and results of other diagnostic tests. Clinical signs reflect the degree of functional impairment and can be used to identify dogs with clinical signs that are strongly or possibly suggestive of PH (Table 1). Attributing clinical signs directly to PH can be challenging or impossible because of underlying disease that may dominate the clinical picture.

### 4.1 Clinical presentation

In dogs, PH occurs as a primary pulmonary vascular disorder or as a sequela to other cardiac, respiratory, or systemic diseases. Signalment, history, and clinical presentation can reflect the underlying cause. Congenital cardiac shunts and developmental lung disease are generally manifested in puppies, whereas acquired cardiac, respiratory, and systemic diseases usually present in adults. Dogs with MMVD and PH usually are older small breed dogs.^18^, ^35^, ^50^, ^51^, ^109^ A breed predisposition for PH secondary to interstitial lung disease has been suggested in West Highland White Terriers^28^ and Pekingese dogs.^99^ Historical data supporting exposure to *Dirofilaria* or *Angiostrongylus* in endemic regions warrant careful scrutiny for these parasites. Exertional syncope, labored respiration or overt respiratory distress, and exercise intolerance are noteworthy clinical signs frequently directly attributable to PH.^40^, ^52^, ^153^, ^154^ Cough is commonly reported but more likely reflects an underlying respiratory disease.^35^ Cardiac auscultation may identify heart murmurs localized to the mitral valve, tricuspid valve or both; a diastolic heart murmur of PR occasionally is detected in patients with severe PH.^52^ A split or loud second heart sound may be auscultated, particularly with severe PH.^154^ A right‐sided systolic murmur,^155^ right‐sided heart failure (eg, a distended abdomen caused by cranial organomegaly or ascites), or both may be detected. Jugular vein distention and pulsation may be present. Cyanosis may occur secondary to primary pulmonary disease or congenital cardiac disease exhibiting right‐to‐left pulmonary‐to‐systemic shunting. Evaluation of the breathing pattern (increased inspiratory effort, increased expiratory effort, mixed inspiratory/expiratory effort, or paradoxical breathing pattern) may provide clues to an underlying airway, parenchymal or pleural cavity disorder, especially when paired with audible noises present with or without the use of a stethoscope.^156^ Pulmonary auscultation may identify muffled sounds, crackles, wheezes, or increased bronchovesicular sounds. Crackles may indicate interstitial lung disease (especially fibrotic lung disease), severe bronchomalacia, or pulmonary edema or exudate.^94^, ^157^, ^158^

## 5 CLASSIFICATION OF PH

In dogs, the prevalence of PH is as yet unknown. There appears to be increased awareness of PH, likely because of increased recognition of clinical signs of PH and underlying disorders that have PH as a comorbid condition and more widespread availability of echocardiography. Historically, LHD (eg, MMVD) had been among the most commonly identified causes of PH.^7^, ^13^, ^18^, ^35^, ^41^, ^42^, ^50^, ^51^, ^52^, ^83^, ^109^, ^152^, ^159^ However, many disorders causing PH may go unrecognized, especially when echocardiography is not performed or when clinical signs attributable to the primary disease dominate the clinical picture.

We propose 6 groups of PH in dogs, loosely following the classification scheme used in humans with modifications^148^ subdivided to reflect similar themes in some or all of the following: cause of PH, clinical presentation, hemodynamic characteristics, pathophysiology, and treatment. Such a classification is intended to improve diagnostic evaluation and optimize therapeutic management of dogs with PH. Finally, classification into well‐defined groups should facilitate future clinical studies.^160^ As in humans, it is expected that evolution of this classification scheme will occur with increased recognition and understanding of underlying conditions.

The proposed clinical classification of PH in dogs comprises the following 6 groups (Table 4): Group 1, pulmonary arterial hypertension (PAH); group 2, LHD; group 3, respiratory disease/hypoxia; group 4, pulmonary emboli/pulmonary thrombi/pulmonary thromboemboli (PE/PT/PTE); group 5, parasitic disease (*Dirofilaria* and *Angiostrongylus*); and group 6, disorders that are multifactorial or with unclear mechanisms. Peer‐reviewed references to support the classification are provided when possible, with the caveat that studies describing a diagnosis of a particular disease in a dog with PH do not necessarily prove that the disease caused PH. Refinement of this classification scheme will likely be needed in the future. When >1 possible cause for PH is present, consideration must be given to the likelihood that each individual underlying cause is contributing to PH. If the contribution to PH from a comorbid condition is minimal, PH should be classified according to the major disease, however, if there potentially are substantial contributions from ≥2 comorbid conditions, they should be placed in group 6, encompassing multifactorial mechanisms. For example, a dog with MMVD without LA enlargement and severe interstitial lung disease would be classified as group 3b1; a dog with stage C MMVD^161^ and severe interstitial lung disease would be classified as group 6a. This discrimination is important because group 6 disorders are likely to require multimodal treatment targeted at each underlying pathologic mechanism. The group 5 classification for PH in dogs represents the largest deviation from the human medical literature, because humans are not affected by PH secondary to *Dirofilaria* or *Angiostrongylus* infection. Table 5 provides a summary of terminology, hemodynamic definitions, and echocardiographic findings of PH with the corresponding proposed clinical classification of PH.

**Table 5:** Terminology, hemodynamic definitions, and echocardiographic findings of PH together with the proposed clinical classification groups of pulmonary hypertension

Terminology	Hemodynamic definition by right heart catheterization used in humans	Echocardiographic findings	Clinical classification group
Precapillary PH	Mean PAP ≥25 mm Hg	No left atrial enlargement	Group 1. Pulmonary arterial hypertension^a^
	PAWP ≤15 mm Hg	At least some findings listed in Table 3 are expected	Group 3. PH due to respiratory disease/hypoxia
	Increased PVR		Group 4. Thromboembolic PH
			Group 5. Parasitic disease
			Group 6. PH with multifactorial and/or unclear mechanisms
Postcapillary PH	Mean PAP ≥25 mm Hg	Left atrial enlargement	Group 2. PH due to left heart disease
	PAWP >15 mm Hg		Group 6. PH with multifactorial and/or unclear mechanisms
Isolated postcapillary PH	DPG <7 mm Hg	Left atrial enlargement	
	PVR not increased		
Combined postcapillary & precapillary PH	DPG ≥7 mm Hg	Left atrial enlargement	
	Increased PVR	At least some findings listed in Table 3 are expected	

Abbreviations: DPG, diastolic pressure gradient (diastolic PAP − mean PAWP); PAP, pulmonary arterial pressure; PAWP, pulmonary arterial wedge pressure; PH, pulmonary hypertension; PVR, pulmonary vascular resistance.

a Congenital cardiac shunts (group 1d1) exhibiting left‐to‐right shunting represents an exception. The PH may be primarily due to increased right heart cardiac output and not increased PVR.

## 6 GUIDELINES FOR DIAGNOSTIC EVALUATION OF DOGS WITH SUSPECTED PH

### 6.1 General comments

Pulmonary hypertension is not a disease per se but rather a hemodynamic and pathophysiologic state present in a wide variety of diseases.^53^, ^154^ Diagnostic testing in suspected cases thus must encompass 2 major goals: (1) to assess the probability of PH using echocardiography and (2) to determine the underlying cause of PH when possible. This information is critical to best guide therapeutic recommendations. In practice, there are 2 pathways of diagnostic evaluation for PH in dogs. The first involves dogs presenting with a spectrum of clinical signs in which diagnostic testing targets identification of a primary cause of disease, with PH considered later in the evaluation. Consensus diagnostic recommendations D1‐D7 below are guidelines for when to pursue echocardiography to assess the probability of PH based on other test results. The second approach involves early assessment of the probability of PH using echocardiography. Because some clinicians identify intermediate or high probability of PH based on suggestive clinical signs, with echocardiography performed early in the diagnostic evaluation (specifically to investigate PH or for other reasons such as a heart murmur), diagnostic algorithms to help determine the underlying cause of PH (ie, groups 1‐6) also are provided (Figures 3, 4, 5, 6, 7).

#### 6.1.1 Consensus recommendations for PH diagnosis

D1. Echocardiography to assess the probability of PH should be considered as an early diagnostic test in dogs with clinical findings suggestive of PH (Table 1)^35^, ^36^, ^51^, ^80^, ^99^, ^109^, ^162^ after physical examination and thoracic imaging rule out another specific disorder not associated with PH.

- *Consensus in 7/7 members of the panel and 5/5 advisory reviewers*

D2. Echocardiography to assess the probability of PH should be considered when thoracic radiography shows evidence of tortuous, blunted, or dilated pulmonary arteries; asymmetric radiolucent lung fields on dorsoventral or ventrodorsal views; patchy, diffuse alveolar infiltrates^40^; a bulge in the region of the pulmonary trunk or right‐sided cardiac enlargement.^52^, ^154^, ^163^

- *Consensus in 7/7 members of the panel and 5/5 advisory reviewers*

D3. Echocardiography to assess the probability of PH should be considered in dogs with clinical signs suggestive of PH (Table 1) and having ascites (modified transudate with noncardiac causes ruled out), a dilated caudal vena cava on point‐of‐care ultrasound (POCUS; diaphragmaticohepatic view) or a dilated caudal vena cava and hepatic veins on abdominal ultrasonography.

- *Consensus in 6/7 members of the panel and 4/5 advisory reviewers*
- Comment: One panelist recommended caution in interpretation of POCUS right‐sided cardiac markers (eg, large caudal vena cava, hepatic venous distension, ascites or gall bladder wall edema) because they had poor utility for discrimination of dogs with and without PH.^164^ One advisory reviewer recommended the removal of POCUS from D3 stating assessment of the caudal vena cava is challenging, subject to intra‐ and inter‐individual variation and complicated by operator experience, patient positioning, and equipment.

D4. Echocardiography to assess the probability of PH may be considered in dogs having spent time in endemic areas and that are, or have a history of, confirmed *Dirofilaria* positivity^165^ or *Angiostrongylus* positivity^162^ with clinical signs (eg, for heartworm disease: coughing, respiratory distress, collapse, anemia, hyperbilirubinemia, exercise intolerance or ascites; for angiostrongylosis: cardiac, respiratory, or neurologic signs or bleeding diathesis) or thoracic radiographic abnormalities (eg, right‐sided heart enlargement; enlarged pulmonary trunk; enlarged, blunted, or tortuous pulmonary arteries; or pulmonary infiltrates) suspected in association with these parasites.

- *Consensus in 7/7 members of the panel and 5/5 advisory reviewers*

Dogs with a condition associated with acute or chronic PE/PT/PTE are at risk for PH. Examples include immune‐mediated hemolytic anemia, spontaneous hyperadrenocorticism, protein‐losing nephropathy, protein‐losing enteropathy, sepsis, neoplasia, and disseminated intravascular coagulation.^115^, ^116^, ^117^, ^118^, ^119^, ^166^, ^167^, ^168^ Additionally, although heartworm disease and angiostrongylosis cause PH by multifactorial mechanisms, an important contributor to increased PAP is parasitic embolism.

D5. Echocardiography to assess the probability of PH should be considered in dogs at high risk^169^ for PE/PT/PTE that have developed clinical signs suggestive of PH particularly with evidence of hypoxemia and thoracic imaging that fails to identify another underlying cause for the respiratory signs.

- *Consensus in 7/7 members of the panel and 5/5 advisory reviewers*

Thoracic computed tomography (CT) is a highly sensitive imaging modality. Especially when incorporated with single or multi‐phase angiography, thoracic CT can provide supportive or definitive evidence for PVDs, pulmonary parenchymal diseases, and PE/PT/PTE, many of which can be associated with PH.

D6. If not already performed, echocardiography to assess the probability of PH should be considered when thoracic CT angiography shows ≥1 of the following:

- A pulmonary trunk‐to‐descending aorta ratio ≥1.4^170^
- Evidence of RA and RV enlargement
- A decreased pulmonary vein‐to‐PA ratio; an increased pulmonary trunk‐to‐ascending aorta ratio, or an increased RV‐to‐LV ratio^92^
- The presence of pulmonary arterial filling defects^171^
- A mosaic attenuation pattern showing small vessels in a region of decreased attenuation (ie, hypoperfusion) on an inspiratory scan that fails to show accentuation of the mosaic attenuation pattern on an expiratory scan (ie, ruling out air trapping)^172^
- Perivascular diffuse nodular to ill‐defined patchy ground‐glass opacity with a global distribution, compatible with pulmonary capillary hemangiomatosis (PCH) or pulmonary veno‐occlusive disease (PVOD)^80^

- *Consensus in 7/7 members of the panel and 5/5 advisory reviewers*
- *Comments: Although measurement of RV* 173, 174 *(and perhaps RA) enlargement evaluated on contrast CT scan is suspected to be a viable metric for PH, peer‐reviewed studies in dogs with PH have not yet been published. Additionally, signalment and physical examination should be used to discriminate dogs with pulmonary valve stenosis that also may have findings a‐c above*.

Although performed uncommonly as a primary diagnostic test for pulmonary disease, lung biopsy specimens may be acquired and submitted for histologic examination and can be very valuable in identifying and characterizing vascular pathology.

D7. Echocardiography to assess the probability of PH should be considered when histologic examination indicates evidence of widespread PVD:

- The use of routine hematoxylin and eosin staining can help elucidate the pathogenesis of PAH. Examples of vascular lesions include arterial or arteriolar medial smooth muscle hypertrophy or hyperplasia, pulmonary arterial intimal hyperplasia or fibrosis, vascular thrombosis, or occlusion, and arterial plexiform lesions. Large numbers of hemosiderophages may indicate pulmonary venous hypertension. Increased numbers of alveolar capillary endothelial cells suggest PCH or potentially severe pulmonary venous hypertension associated with markedly increased LA pressures.
- Verhoeff‐Van Gieson staining is a valuable additional technique that assists in distinguishing arteries and veins and can be very helpful in identifying affected veins in PVOD.

•*Consensus in 7/7 members of the panel and 5/5 advisory reviewers*
•*One panelist commented that adequately large lung biopsy specimens and multiple (>2) lung biopsies may be needed to characterize the underlying disease process. The absence of lesions especially in small biopsy specimens or in end‐stage tissue (ie, fibrosis) may not allow the elimination of vascular pathology as a possibility. Additionally, the pathologist evaluating the lesions must be knowledgeable about PVDs*.

## 7 TREATMENT

Treatment of PH can be subdivided into strategies to decrease the risk of progression or complications (treatment consensus statements T1a‐e), recommendations to target underlying diseases or factors contributing to PH (treatment consensus statements T2‐T12), and PH‐specific treatments (treatment consensus statements T13‐T24).

Interpretation of therapeutic recommendations is dependent on the level of evidence, degree of clinical impairment, and echocardiographic probability of PH. The strength of the recommendation is higher when the primary literature is available regarding treatment of dogs with spontaneous PH (aside from single case reports) or, in the absence of these data, strong expert opinion, and the treatment statements are worded as “recommended.” When recommendations are extrapolated from humans, canine models, or weaker anecdotal experience of experts, the treatment statements are worded as “may be considered.” As reviewed in Tables 1 and 2, clinical findings are stratified as strongly or possibly suggestive of PH and echocardiographic evidence of PH as low, intermediate, or high probability.

### 7.1 Strategies to decrease the risk of progression or complications of PH

T1. Several guidelines yet untested in randomized clinical trials, seem prudent, especially in dogs with high probability of PH:

- Exercise restriction
- Prevention of contagious respiratory pathogens using vaccination^175^ and parasitic disease (eg, *Dirofilaria* and *Angiostrongylus*) control using chemoprophylaxis in endemic areas
- Avoidance of pregnancy (because of potential to exacerbate PH and because of the possibility of transmission of genetic contributors)
- Avoidance of high altitude and air travel
- Avoidance of nonessential wellness procedures (eg, dental cleanings) and elective surgery requiring general anesthesia

- *Consensus in 7/7 members of the panel and 5/5 advisory reviewers*

### 7.2 Recommendations to target underlying diseases or factors contributing to PH

Long‐term supplemental oxygen has yet to be evaluated as supportive treatment using randomized clinical trials in people with PH but generally is recommended.^12^ A recent large observational study showed benefit in PAH.^176^ At home, oxygen treatment is feasible in dogs and could be considered, especially if there appears to be a positive clinical response. Additional studies are warranted.

#### 7.2.1 Group 1 PAH

For the majority of group 1 dogs, there is no effective primary treatment and PH‐specific treatment is the major means of management (see T13‐T15 below).

T2. Shunt closure or occlusion is recommended in dogs in group 1d1, provided the shunt is hemodynamically relevant (ie, cardiac remodeling is present or likely to develop) and the shunt is exclusively from left to right or becomes so upon administration of pulmonary vasodilators.

T3. It is recommended that dogs in group 1d1 exhibiting right‐to‐left shunting and having erythrocytosis and clinical signs be treated by periodic phlebotomy, typically with fluid replacement.^54^ Hydroxyurea can be considered as an alternative to decrease red cell volume.^177^

- *Consensus in 7/7 members of the panel and 5/5 advisory reviewers*

#### 7.2.2 Group 2 PH secondary to LHD

Treatment strategies for targeting the underlying disease in group 2 patients are centered around identifying and, if possible, reversing the cause of LHD, decreasing postcapillary PH (ie, lowering LA pressure) and, if present, treating heart failure. Management strategies for specific LHD and left‐sided heart failure (LHF) are beyond the scope of this consensus statement. However, readers are encouraged to consult the ACVIM consensus guidelines for the management of MMVD,^161^ which includes management strategies for LHF and preclinical MMVD treatment, and the veterinary literature evaluating pharmacotherapy to delay the onset of heart failure in dogs with common LHD such as MMVD^178^, ^179^ and dilated cardiomyopathy.^180^

T4. Because dogs with PH secondary to LHD, by definition, have postcapillary PH (with or without pre‐PH), the use of phosphodiesterase 5 inhibitors (PDE5i) is not recommended as first line treatment.

- *Consensus in 7/7 members of the panel and 5/5 advisory reviewers*

#### 7.2.3 Group 3 PH secondary to respiratory disease, hypoxia, or both

Group 3 dogs have diverse respiratory diseases, and an exhaustive discussion of specific treatments is beyond the scope of this consensus statement. Treatment of the underlying respiratory disorder should decrease the severity of clinical signs, improve the perceived quality of life, and attenuate, halt, or delay the progression of pathology leading to further impairment in respiratory function.

T5. General strategies of value that are recommended include weight loss in obese patients, environmental modifications to improve air quality and optimize humidity, and reduction of recognized triggers of clinical signs. In obese patients, weight loss can decrease clinical signs by increasing thoracic wall compliance and decreasing extrathoracic and intra‐abdominal adipose tissue.^181^ Although not documented in dogs to date, morbid obesity may cause severe but reversible PH in people,^182^, ^183^ underscoring its importance in the general management strategy. Environmental changes, reduction of specific triggers of barking, anxiety, and excitement, as well as the use of a harness instead of a neck collar can help decrease the stimulus to cough.^184^

- *Consensus in 7/7 members of the panel and 5/5 advisory reviewers*

T6. In group 3a disorders, recommended treatment is primarily symptomatic and includes cough suppression, sedation, oxygen supplementation, and, when present, control of secondary infection and inflammation. Specific examples may include, but are not limited to, glucocorticoids, opioids or other sedatives, antimicrobials, and antitussives.^181^, ^185^, ^186^, ^187^, ^188^ For management of severe tracheal collapse, placement of an intraluminal stent can be considered.^189^, ^190^

- *Consensus in 7/7 members of the panel and 5/5 advisory reviewers*

T7. The group 3b disorders are diverse, some with specific treatments and some in which viable treatment options do not exist.

- Within group 3b1, fibrotic lung disease to date has no effective treatments, likely reflecting end‐stage lesions and lack of understanding of specific triggers.^94^, ^158^ In some cases of fibrotic lung disease, PO or inhaled corticosteroids may relieve cough, particularly in the presence of concurrent bronchial changes.^191^, ^192^ Dogs with cryptogenic organizing pneumonia receiving early and aggressive treatment with immunosuppressive doses of glucocorticoids may have a good prognosis.^94^ Whole lung lavage has been described to treat pulmonary alveolar proteinosis.^193^ Corticosteroids are the primary treatment for eosinophilic lung disease.^95^, ^185^, ^186^
- In dogs with group 3b2 disorders in which infection underlies pathology, appropriate antimicrobials are recommended. For example, pneumocystis pneumonia should be treated with high‐dose trimethoprim‐sulfonamide with or without an anti‐inflammatory dose of corticosteroids.^102^, ^194^
- In dogs with group 3b3 diffuse pulmonary neoplasia, consultation with a veterinary oncologist is recommended because options are limited and for most cancers (aside from lymphoma), and prognosis is grave.

- *Consensus in 7/7 members of the panel and 5/5 advisory reviewers*

T8. In group 3c, dogs with brachycephalic obstructive airway syndrome (BOAS) and other causes of upper airway obstruction that have been less clearly documented to cause PH, early recognition and medical or surgical management is recommended. In BOAS, the upper airway obstruction components that can be surgically corrected (eg, elongated soft palate, stenotic nares, aberrant rostral and caudal turbinates, everted saccules) should be surgically treated early in life to minimize the progression of clinical signs and avoid possible development of PH.^195^, ^196^, ^197^, ^198^, ^199^ Although the link to development of PH is unclear, concurrent management of alimentary tract disease contributing to respiratory disease is prudent, even in the absence of overt dysphagia, vomiting, and regurgitation.^198^, ^200^

- *Consensus in 7/7 members of the panel and 5/5 advisory reviewers*

#### 7.2.4 Group 4 PH secondary to PE/PT/PTE

Treatments for underlying causes of PE/PT/PTE are beyond the scope of this consensus statement and are reviewed elsewhere.^201^ A recent consensus statement on the rationale use of antithrombotics is relevant.^202^

T9. In dogs with PH caused by suspected or confirmed PE/PT/PTE, prompt treatment with antithrombotic agents should be instituted. Heparin (low molecular weight or unfractionated) and PO direct anticoagulants (eg, rivaroxaban, apixaban) may be preferred over PO antiplatelet agents (eg, clopidogrel, aspirin).

- *Consensus in 7/7 members of the panel and 5/5 advisory reviewers*

T10. In dogs with PH caused by acute PE/PT/PTE and having overt RV dilatation and systolic dysfunction associated with systemic hypotension and collapse, immediate use of systemic or local tissue plasminogen activator (with or without concurrent endovascular or surgical thrombectomy) may be considered, with an understanding of the potential risks and appropriate access to intensive 24‐hour monitoring.

- *Consensus in 7/7 members of the panel and 5/5 advisory reviewers*

#### 7.2.5 Group 5 PH secondary to parasitic disease (Dirofilaria or Angiostrongylus infection):

T11. The reader is referred to guidelines for management of heartworm disease^165^ and angiostrongylosis.^203^

- *Consensus in 7/7 members of the panel and 5/5 advisory reviewers*

#### 7.2.6 Group 6 PH with multifactorial or unclear mechanisms

Treatment of the group 6a disorders should focus on identifying and addressing individual pathology contributing to PH when possible (see above recommendations).

T12. When feasible in group 6b dogs, medical, endovascular, or surgical treatment to address the compressive mass lesion (eg, treatment of blastomycosis, radiation therapy for heart base masses, intravascular stents) is recommended.

- *Consensus in 7/7 members of the panel and 5/5 advisory reviewers*

### 7.3 PH‐specific treatment

Excessive pulmonary arterial vasoconstriction secondary to a variety of pulmonary arterial endothelial insults develops via the nitric oxide, endothelin, or prostacyclin pathways.^204^ In people, recommendations for PH‐specific treatment focus on maximizing vasodilatory response by targeting multiple pathways concurrently. In dogs, initial simultaneous targeting of all pathways is uncommon because of the lack of evidence, feasibility, cost, and quality of life issues with repeated medication administration. In dogs, the first‐line treatment of PH consists of PDE5i that specifically target and augment the vascular nitric oxide pathway. The PDE5i are intended to target pre‐PH by decreasing PVR. Dose escalation of PDE5i or other PH‐specific medications may be considered if the patient becomes refractory and if severe clinical signs warrant more aggressive treatment.

Most of the peer‐reviewed veterinary medical literature regarding dogs with PH has evaluated the PDE5i, sildenafil.^40^, ^42^, ^43^, ^52^, ^80^, ^84^, ^205^, ^206^, ^207^ These studies suggest benefit with improvement of clinical signs,^52^ quality of life,^52^, ^84^, ^207^ exercise capacity,^84^ and decreased echocardiographically‐estimated PAP compared with baseline,^42^, ^84^, ^207^ but TRV might not decrease after PDE5i treatment despite observed clinical benefits.^52^ This outcome may occur because pulmonary blood flow might increase as PVR decreases, thus resulting in little change in PAP. Additionally, aforementioned limitations of echocardiography to estimate PAP also may play a role. Sildenafil has a short half‐life, ideally necessitating q8 hour dosing,^208^, ^209^ which represents a disadvantage. Rectal administration of sildenafil can be considered when PO dosing is not feasible.^209^ More recently, tadalafil has emerged as an appealing alternative^210^ with a longer half‐life, allowing for q24h dosing, improved compliance and, in some cases, lower cost. In a randomized double‐blinded study comparing sildenafil and tadalafil in dogs with PH, PDE5 inhibition was safe and improved quality of life without demonstrating superiority of 1 PDE5i over the other.^210^

Treatment strategies for PH are highly dependent on cause and chronicity of PH. Some PH‐specific treatments (eg, pulmonary artery vasodilators such as PDE5i) might lead to acute pulmonary edema in some dogs with PH. Therefore, pulmonary artery vasodilators in some specific situations such as dogs with PH associated with congenital cardiac shunts (group 1d1) or secondary to LHD (group 2) warrants caution. In dogs with PH associated with congenital cardiac shunts, increased PAP can be caused primarily by increased blood flow through the pulmonary vasculature, reactive pulmonary vasoconstriction, or may occur secondary to PVD. Often it is difficult using echocardiography to discern the relative impact of each of these factors on estimated PAP. Dogs in group 1d1 without substantially increased PVR exhibit left‐to‐right (systemic‐to‐pulmonary) shunting and will benefit from closure or occlusion of the shunt rather than a pulmonary artery vasodilator. Dogs in group 1d1 with increased PVR might benefit from a pulmonary artery vasodilator, particularly if they are exhibiting bidirectional or right‐to‐left (pulmonary‐to‐systemic) shunting and erythrocytosis. Closure will not be possible if PVR exceeds systemic vascular resistance. In some cases, shunt flow may reverse (ie, become left‐to‐right) after administration of a pulmonary artery vasodilator, thus permitting safer closure or occlusion of the shunt.^55^ However, a pulmonary artery vasodilator might induce pulmonary edema in some dogs in this scenario if they have “reactive” or “responsive” pulmonary arteries (or arterioles) and substantial irreversible PVD has not developed.

Similarly, dogs in group 2 may (C‐PH) or may not (Ipost PH) have increased PVR (Table 5). In addition to treatment specifically targeting the LHD and LHF, some dogs with C‐PH might benefit from a PDE5i in an attempt to alleviate clinical signs. However, similar to dogs in group 1d1, their vascular reactivity or responsiveness to a pulmonary vasodilator is difficult to predict, and pulmonary edema also may ensue. The mechanism of inducing pulmonary edema is similar in dogs in groups 1d1 and 2. In both situations, a pulmonary artery vasodilator might increase right heart cardiac output, acutely increasing pulmonary venous return to the LA and subsequently increase LA and thus pulmonary venous and capillary pressures, resulting in pulmonary edema. In scenarios in which a PDE5i is initiated (see below), close monitoring for development of pulmonary edema is strongly recommended. Ideally, such monitoring should be done in a veterinary hospital. Resting or sleeping respiratory rate and effort should be closely monitored. Pulmonary edema should be ruled out (eg, by thoracic radiography) if sleeping or resting respiratory rates are consistently >30‐40 breaths/min^211^, ^212^ or if respiratory distress is observed. Because of the risk of inducing pulmonary edema in this context, some clinicians advise starting with a conservative dosage of pulmonary vasodilator medications (eg, sildenafil 0.5 mg/kg PO q8h).

Unless otherwise stated, consensus recommendations for PH‐specific treatment below assume that clinical findings suggestive of or associated with PH are apparent (Table 1) and the proposed clinical definition of PH has been fulfilled, that is, intermediate or high probability of PH is present (Tables 2, 3).

The panel does not advocate PH‐specific treatment in dogs without clinical signs or findings suggestive of PH.

#### 7.3.1 Group 1 PAH

Insufficient data are available in the veterinary medical literature to follow consensus recommendations for humans. In treatment‐naive humans, management begins with acute vasoreactivity testing (generally using a short acting vasoactive agent such as nitric oxide) to determine the likelihood of response to initial therapeutic trials.^213^ Empirical treatment is advocated in dogs.

T13. A PDE5i is recommended for group 1a, 1b, and 1c because without treatment the prognosis is guarded to grave and no specific treatment is available for underlying disease. Anecdotal experience suggests a subpopulation of dogs with suspected group 1a‐c disorders may have a good clinical response to sildenafil.

- *Consensus in 7/7 members of the panel and 5/5 advisory reviewers*

Dogs with group 1 PAH associated with occlusive cellular or fibrotic vascular occlusive lesions or both (ie, group 1d2, 1d3, and 1′) may have a lesser contribution from defects in vasomotor tone. Once PH has been diagnosed, patients with these disorders tend to have short survival periods, no cure and poor response to typical PH‐specific treatments. A lack of clinical trials assessing treatment response is challenging because these diseases are rare, advanced, and frequently lack a definitive antemortem diagnosis.

T14. Using PDE5i in dogs with group 1’ disorders may result in fatal acute pulmonary edema as seen in some humans; this may occur because higher blood flows are not accommodated by the fixed downstream obstruction in the veins, capillaries, or both. Although insufficient evidence of a similar phenomenon exists in the veterinary medical literature,^80^ the recommendation is that PDE5i be initiated in the hospital in dogs with known or suspected PVOD and PCH, with close monitoring for development of acute pulmonary edema. The drug should be withdrawn immediately if this complication occurs. Because PVOD and PCH are most likely to be definitively diagnosed postmortem, a high index of suspicion antemortem will be required to maintain appropriate vigilance.

- *Consensus in 7/7 members of the panel and 5/5 advisory reviewers*

In dogs in group 1d1 exhibiting right‐to‐left (pulmonary‐to‐systemic) shunting, morbidity and mortality generally are thought to be linked to effects of chronic hypoxemia and erythrocytosis rather than heart failure. The use of PDE5i may attenuate both PH and clinical signs and help manage secondary erythrocytosis.^206^ In the panelists' experience, survival of dogs with right‐to‐left shunting is highly variable, and untreated dogs have been observed to remain free of clinical signs and survive for prolonged periods of time.

T15. A PDE5i may be considered in dogs in group 1d1 exhibiting bidirectional or right‐to‐left shunting in an attempt to improve clinical signs and help manage erythrocytosis. In this scenario, hematocrit might serve as an objective variable to monitor response to the PDE 5i. In‐hospital monitoring when PDE 5i treatment is initiated is advisable for the reasons discussed above.

- *Consensus in 7/7 members of the panel and 5/5 advisory reviewers*

#### 7.3.2 Group 2 PH secondary to LHD

Treatments for pre‐PH (ie, pulmonary artery vasodilators) in people with LHD are controversial and not routinely advised because of the risk of adverse events and lack of compelling data showing benefit.^214^ Although addressing the underlying LHD or LHF is most important, PH‐specific treatment in dogs with group 2 disease may be considered as adjunctive in selected cases in an attempt to alleviate clinical signs. The following recommendations assume cardiogenic pulmonary edema has been ruled out (eg, by thoracic radiography) because a PDE5i should be administered only to dogs free of acute or decompensated LHF (cardiogenic pulmonary edema).

T16. Heart failure medications (HFM) and a PDE5i are recommended for dogs with clinical (eg, jugular venous distension, fluid wave on abdominal palpation, or auscultable pleural fluid line) and ultrasonographic (abdominal or pleural effusion without another cause along with RA enlargement, caudal vena caval distension, hepatic venous distension or hepatomegaly) evidence of right‐sided heart failure.

- *Consensus in 7/7 members of the panel and 5/5 advisory reviewers*

T17. Addition of a PDE5i may be considered in dogs with exertional syncope without another identifiable cause that have failed to respond to other treatments for preclinical LHD (eg, pimobendan).

- *Consensus in 7/7 members of the panel and 5/5 advisory reviewers*

T18. A PDE5i may be considered for dogs with a high probability of PH with compensated LHF (ie, dogs previously diagnosed with LHF and on HFM but that do not currently have pulmonary edema) that develop cardiogenic ascites. Treatment should be carried out by up‐titration of HFM to offset the potential risk of inducing pulmonary edema.

- *Consensus in 7/7 members of the panel and 5/5 advisory reviewers*

T19. A PDE5i may be considered for dogs with compensated LHF and a high probability of PH (ie, dogs previously diagnosed with LHF and on HFM but that do not currently have pulmonary edema) that develop exertional syncope without another identifiable cause. A thorough diagnostic evaluation is advised to rule out alternative causes of syncope (eg, ECG, ambulatory ECG monitoring, or both to exclude bradyarrhythmias or tachyarrhythmias as potential causes for syncope).

- *Consensus in 7/7 members of the panel and 5/5 advisory reviewers*

#### 7.3.3 Group 3 PH secondary to respiratory disease, hypoxia, or both

In people, for the same reasons noted for group 2 PH, the use of PH‐specific treatments for group 3 PH are not routinely recommended.^214^ In contrast, in dogs with group 3 PH, there may be benefit to additional PH‐specific treatment during or after treatment for underlying pulmonary disease. In a recent study of dogs with diverse causes of group 3 PH, administration of PDE5i was the only independent predictor of survival in a multivariable analysis.^91^

T20. A PDE5i is recommended in group 3 dogs.^40^, ^42^, ^52^, ^84^, ^205^, ^210^

- *Consensus in 7/7 members of the panel and 5/5 advisory reviewers*

#### 7.3.4 Group 4 PH secondary to PE/PT/PTE

In a canine model of acute pulmonary embolism, sildenafil decreased mean PAP and blunted decreases in PaO_2_.^215^ Sildenafil has been used rarely in people with acute pulmonary embolism leading to PH, despite showing benefit.^216^, ^217^, ^218^ The PH classification scheme used in humans focuses on chronic thromboembolic PH, in which PH‐specific treatments are indicated in patients who are not surgical candidates for endarterectomy, if preoperative hemodynamic stabilization is needed and if signs persist or recur after surgery.^214^ In dogs with acute and chronic group 4 PH, additional PH‐specific treatment can be considered during or after treatment directly targeting underlying causes of PE/PT/PTE.

T21. Although only experimental evidence in a canine model supports the use of sildenafil in acute PE, PDE5i may be considered in group 4a patients (acute massive PE/PT/PTE) with overt RV dilatation and RV systolic dysfunction.

- *Consensus in 7/7 members of the panel and 5/5 advisory reviewers*

T22. A PDE5i is recommended in addition to anticoagulant treatment for group 4b patients (chronic PE/PT/PTE).^40^, ^42^

- *Consensus in 7/7 members of the panel and 5/5 advisory reviewers*

#### 7.3.5 Group 5 PH secondary to parasitic disease (Dirofilaria or Angiostrongylus infection)

T23. The 2018 American Heartworm Society guidelines do not provide recommendations for PH‐specific treatment. No peer‐reviewed studies have investigated PH‐specific treatment solely in dogs with heartworm disease. Treatment with a PDE5i may be considered for dogs with *Dirofilaria immitis* infection.^210^, ^219^

- *Consensus in 7/7 members of the panel and 5/5 advisory reviewers*

T24. In a small retrospective study of dogs with angiostrongylosis and PH, treatment with sildenafil did not impact survival.^162^ However, because moderate to severe PH is recognized in approximately 15% of dogs with *Angiostrongylus vasorum* infection, and these dogs have a worse prognosis than those without PH,^162^ treatment using a PDE5i may be considered.

- *Consensus in 7/7 members of the panel and 5/5 advisory reviewers*

#### 7.3.6 Group 6 PH with multifactorial or unclear mechanisms

Because group 6a disorders have multifactorial mechanisms, arise from independent comorbid conditions, or both, it is critical that treatment targets each underlying pathologic mechanism as outlined above (treatment recommendations T2‐T12). Because comorbid conditions may independently fall into groups 1‐5, the clinician also should be familiar with when PH‐specific treatments are recommended within each group (treatment recommendations T13‐T24) and integrate this information to formulate a final treatment plan.

#### 7.3.7 Additional PH‐specific treatments

In dogs with progressive disease or in those failing to respond when dose‐escalating PH‐specific treatments described above have been used, alternative or adjunctive treatments may be sought (Box 1). Insufficient evidence is available at this time to recommend other treatments.

BOX 1 **Unproven alternative or adjunct therapies that might be considered for use in dogs with pulmonary hypertension**

**Table:** 

**Pimobendan:** Pimobendan is an oral PDE3i with positive inotropic and systemic vasodilatory properties. It has been shown to improve RV systolic function following a single oral dose in healthy dogs.^26^ Although pimobendan has been suggested as a treatment for PH in general,^24^, ^83^, ^105^, ^205^, ^220^ to date, there is no direct or clear evidence of its beneficial effects on pre‐PH. Previously reported improvements in estimated PAPs in dogs with MMVD might be related to its beneficial effect on lowering LA pressure^221^ and thus targeting postcapillary PH. Further study is needed to help clarify pimobendan's role in pre‐PH. Thus, the panel does not advocate for or against the use of pimobendan as adjunct treatment in dogs with pre‐PH.
**Milrinone:** Milrinone is an IV PDE3i. It has both PA vasodilating and positive inotropic properties. In experimental canine PH, milrinone improved RV function^222^ and decreased mean PAP.^223^
**Tyrosine kinase inhibitors (eg, toceranib, imatinib):** Tyrosine kinase inhibitors (TKI) result in PA vasodilation by inhibiting the activation of platelet derived growth factor by impeding phosphorylation of the platelet‐derived growth factor receptor tyrosine kinase. In people, specific TKI are effective at improving refractory PH^224^, ^225^, ^226^, ^227^, ^228^ but serious adverse events are common.^228^ In dogs, a single study demonstrated imatinib reduced PAP in dogs diagnosed with PH secondary to LHD.^229^ Paradoxically, some TKI can induce PH in humans.^230^ Consideration of and monitoring for contraindications and adverse events are indicated.
**L‐arginine:** L‐arginine is an amino acid that is essential, in conjunction with oxygen, to the production of NO. Oral administration increases surrogate markers of NO in healthy dogs.^231^ Although no clinical studies in dogs have demonstrated the benefits of L‐arginine in clinical patients, 1 study in experimental canine acute PTE showed L‐arginine and sildenafil together were not more beneficial than sildenafil alone.^232^
In dogs, there is insufficient information in the literature and anecdotal experience with other PH‐specific therapies used in humans (eg, calcium channel blockers, endothelin antagonists, prostanoids, soluble guanylate cyclase stimulators, etc). No recommendations on the use of these medications can be made at the current time.

## 8 GUIDELINES FOR LONG‐TERM MONITORING

Clinical experience suggests the prognosis of dogs with PH is variable and related to the cause of PH. Unless a reversible cause of PH is identified, dogs with a high probability of PH are likely to experience a worse prognosis than dogs with same disease but with a low probability of PH. This was demonstrated in a large retrospective study of dogs with MMVD, likely the most common cause of PH in dogs.^82^ Right‐sided heart failure secondary to PH also likely has a worse prognosis. Factors such as cost of care, client financial resources, and commitment to care, and the client's and attending clinician's perception of PH (and its underlying cause) can affect survival times, which makes it challenging to prognosticate for individual dogs with PH. Regardless of the cause of PH, once PH‐specific treatment is initiated, patients should be monitored for improvement, static condition, or progression using consensus recommendations for monitoring given below. Additionally, whenever possible, any identified underlying disorder should be addressed and monitored simultaneously. Recommendations for monitoring underlying disorders are beyond the scope of this consensus statement, and guidelines to supplement specific recommendations for monitoring underlying disorders (Box 2) should be sought elsewhere.

BOX 2 **Specific considerations for monitoring underlying disorders**

**Table:** 

**Group 1 PAH**: Dogs in group 1a, 1b, 1c, 1d2, and 1d3 that have an antemortem diagnosis should follow consensus monitoring recommendations above. In group 1d1, some congenital heart diseases may require additional monitoring to guide treatment (eg, Eisenmenger's physiology should have a PCV or hematocrit when clinical signs are present to guide phlebotomy and administration of fluids or hydroxyurea).^54^, ^177^ Similar to dogs in group 1d1 and group 2, dogs in group 1’ might also develop acute pulmonary edema following administration of a pulmonary artery vasodilator. Thus, if diagnosed antemortem (uncommon), appropriate monitoring (as previously discussed) is warranted following administration of pulmonary artery vasodilators.
**Group 2 PH secondary to left‐sided cardiac disease**: In group 2 dogs, monitoring recommendations often depend on where in the stage of heart disease the dog is classified. If a dog develops postcapillary PH, the cardiac disease is more advanced. In all group 2 dogs, the main points to monitor are the status of heart failure (primarily via thoracic radiography), and renal function and electrolyte status (both assessed by a biochemical profile). Echocardiography may be indicated to assess for adverse complications of left‐sided cardiac disease that can result in decompensation of the patient, such as ruptured chordae tendineae, pericardial effusion secondary to an atrial tear, or the development of an acquired atrial septal defect secondary to a tear of the interatrial septum. For dogs with an infectious component to the left‐sided cardiac disease, as in group 2a2 and 2c1b, monitoring for changes in the valve leaflets or chamber walls may be accomplished with echocardiography. Serial analysis of cardiac troponin I might be useful in monitoring dogs with a presumptive diagnosis of myocarditis. The reader is referred to the ACVIM consensus statement on MMVD in dogs^161^ and a comprehensive review of DCM in dogs^233^ for specific monitoring recommendations for the underlying LHD.
**Group 3 PH secondary to respiratory disease, hypoxia, or both**: In many of the group 3 disorders (eg, 3a, 3b1b, 3b1e, 3b2, 3c), clinical signs can guide titration of medications. In case of acute disease exacerbation or development of new clinical signs, repeating some of the initial diagnostics (eg, imaging) may be warranted. Serial thoracic radiography can be performed to assess for improvement using glucocorticoids in dogs with group 3b1b and 3b1e disorders and in dogs receiving antimicrobials in group 3b2. Dogs in group 3b1, particularly those with fibrotic lung disease (3b1a) and chronic progressive ILDs (3b1d), can be monitored using measurement of arterial blood gas (or pulse oximetry) or a 6MWT.^234^, ^235^
**Group 4 PH secondary to PE/PT/PTE**: Duration of antithrombotic treatment depends if the underlying condition(s) have resolved (in which case antithrombotic treatment should be discontinued following resolution of the thrombus) or are persistent (in which case antithrombotic treatment should continue indefinitely).^169^ The reader is referred to recent consensus guidelines for specific anti‐thrombotic monitoring and weaning recommendations.^169^
**Group 5 PH secondary to parasitic disease (heartworm or angiostrongylus infection)**: Confirmation of efficacy of *Dirofilaria immitis* adulticidal treatment is most reliably performed using heartworm antigen testing.^165^ In dogs with angiostrongylosis, monitoring can be performed by using antigen‐based assays or molecular techniques on blood samples.^236^
**Group 6 PH with multifactorial or unclear mechanisms**: For group 6b dogs receiving targeted treatment, thoracic imaging every 1‐3 months to evaluate the size of the mass compressing the pulmonary arteries may be considered, with cross‐sectional imaging (CT) likely more sensitive to detect changes in mass size than thoracic radiography.

A patient‐specific and goal‐oriented strategy for treatment is recommended and necessitates careful monitoring with adjustment of medications based on the magnitude of response and adverse effects. Clinical improvement, thoracic radiography, pulse oximetry, and arterial blood gases represent the most useful serial diagnostic tests. Other means of monitoring include echocardiography, N terminal pro‐B‐type natriuretic peptide, 6‐minute walk test (6MWT), and voluntary activity monitors. In people, the magnitude of PAP correlates poorly with clinical signs and outcome and is not recommended as a sole variable to guide therapeutic decisions.^214^ Similarly in dogs, improvement in echocardiographic indices with PDE5i administration is sometimes^40^, ^42^, ^84^ but not always^52^, ^206^, ^210^ identified. In comparison, the 6MWT in people is a simple, reproducible, and standardized means of assessing exercise capacity^237^ and is a critical primary endpoint used in most randomized controlled trials. In dogs, the 6MWT has been used to discriminate healthy dogs from dogs with respiratory disease.^234^, ^235^ It also has been performed in dogs with tricuspid regurgitation and MMVD.^10^ Voluntary activity monitors^84^, ^210^ allow assessment of exercise capacity for longer periods of time (days to weeks) but are ethically challenging to use in comparing baseline to treatment effect in patients requiring imminent treatment.

M1. It is recommended that clinical assessments (degree and direction of change in exercise tolerance, syncope, right‐sided heart failure, and respiratory rate and effort) play the major role in evaluation of response to treatment and need for escalation of treatment. Baseline assessments should be made to allow evaluation of treatment response. In stable patients, clinical evaluation may be considered 2 weeks after starting or changing PH‐specific treatment, every 3‐6 months thereafter, and at any time when exacerbation of clinical signs occurs. In unstable patients, clinical evaluation will be dictated by the patient's needs. Questionnaires to assess owner‐reported quality of life (eg, functional evaluation of cardiac health^238^) also may be beneficial.

- *Consensus in 7/7 members of the panel and 5/5 advisory reviewers*

M2. Although echocardiographic indices of PH may not always correlate with clinical improvement or decompensation, echocardiography may be repeated at the clinician's discretion. Regularly repeated echocardiograms in clinically stable patients may not be necessary in all dogs with PH.

- *Consensus in 7/7 members of the panel and 5/5 advisory reviewers*

M3. Other complementary diagnostic tests may be considered to guide treatment including thoracic imaging, pulse oximetry, arterial blood gases, N terminal pro‐B‐type natriuretic peptide, 6MWT, and activity monitors. Although it is recognized that a panel of data reflecting patient status is likely to be superior to any single outcome variable, the optimal means to evaluate patient status is, as yet, unclear. Complementary diagnostic tests may be considered 2 weeks after starting or changing PH‐specific treatment, or in patients with underlying disease impacting clinical signs, based on the clinician's discretion.

- *Consensus in 7/7 members of the panel and 5/5 advisory reviewers*

## CONFLICT OF INTEREST DECLARATION

Carol Reinero received donation of medication (tadalafil) for an intramurally funded clinical trial comparing efficacy of sildenafil and tadalafil in dogs with pulmonary hypertension (2017‐2018). Brian Scansen has received speaking fees and travel expense reimbursement from Boehringer Ingelheim Animal Health. All other authors have no declared conflicts of interest.

## OFF‐LABEL ANTIMICROBIAL DECLARATION

Authors declare no off‐label use of antimicrobials.

## INSTITUTIONAL ANIMAL CARE AND USE COMMITTEE (IACUC) OR OTHER APPROVAL DECLARATION

Authors declare no IACUC or other approval was needed.

## HUMAN ETHICS APPROVAL DECLARATION

Authors declare human ethics approval was not needed for this study.
